# Comparative efficacy between atorvastatin and rosuvastatin in the prevention of cardiovascular disease recurrence

**DOI:** 10.1186/s12944-019-1153-x

**Published:** 2019-12-11

**Authors:** Sofía Perez-Calahorra, Martin Laclaustra, Victoria Marco-Benedi, Xavier Pinto, Rosa M. Sanchez-Hernandez, Núria Plana, Emilio Ortega, Francisco Fuentes, Fernando Civeira

**Affiliations:** 1 Lipid Unit, Hospital Universitario Miguel Servet, IIS Aragon, CIBERCV, Zaragoza, Spain; 2 Lipid and Vascular Risk Unit. Internal Medicine Service, CIBEROBN, Hospital de Bellvitge, Hospitalet de Llobregat, Spain; 3 Lipid Unit, Endocrinology Department, Hospital Universitario Insular de Gran Canaria, Instituto Universitario de Investigaciones Biomédicas y Sanitarias, Universidad de Las Palmas de Gran Canaria, Las Palmas de Gran Canaria, Spain; 4 Unitat de Medicina Vascular i Metabolism, Hospital Universitari Sant Joan, Institut d’Investigació Sanitària Pere Virgili (IISPV), CIBERDEM, Reus, Tarragona, Spain; 5 Lipid Unit, Endocrinology and Nutrition Service, Institut d’Investigacions Biomèdiques August Pi Sunyer, CIBEROBN, Hospital Clínic, Barcelona, Spain; 6 Lipid Unit, Instituto Maimónides de Investigación Biomédica de Córdoba (IMIBIC), CIBEROBN, Universidad de Córdoba, Hospital Universitario Reina Sofía, Córdoba, Spain; 7 Universidad de Zaragoza, Zaragoza, Spain

**Keywords:** Rosuvastatin, Atorvastatin, Secondary prevention, High-potent statin

## Abstract

**Background:**

There is no randomized clinical trials with recurrence of atherosclerotic cardiovascular disease (ASCVD) as a major outcome with rosuvastatin. In order to analyze potential differences in the clinical response to atorvastatin and rosuvastatin in secondary ASCVD prevention, we have analyzed the clinical evolution of those subjects of the Dyslipemia Registry of the Spanish Society of Arteriosclerosis (SEA) who at the time of inclusion in the Registry had already suffered an ASCVD.

**Methods:**

This observational, retrospective, multicenter, national study was designed to determine potential differences between the use of atorvastatin and rosuvastatin in the ASCVD recurrence. Three different follow-up start-times were performed: time of inclusion in the registry; time of first event if this occurred after 2005, and time of first event without date restriction.

**Results:**

Baseline characteristics were similar between treatment groups. Among atorvastatin or rosuvastatin users, 89 recurrences of ASCVD were recorded (21.9%), of which 85.4% were coronary. At the inclusion of the subject in the registry, 345 participants had not suffered a recurrence yet. These 345 subjects accumulated 1050 person-years in a mean follow-up of 3 years. Event rates were 2.73 (95% CI: 1.63, 4.25) cases/100 person-years and 2.34 (95% CI: 1.17, 4.10) cases/100 person-years in the atorvastatin and rosuvastatin groups, respectively. There were no statistically significant differences between the two groups independently of the follow-up start-time.

**Conclusions:**

This study does not find differences between high doses of rosuvastatin and atorvastatin in the recurrence of ASCVD, and supports their use as clinically equivalent in secondary prevention of ASCVD.

## Background

Reduction of cholesterol transported in low-density lipoproteins (LDLc) is one of the mainstays of atherosclerotic cardiovascular disease (ASCVD) prevention, since multiple studies have demonstrated the causal role of LDLc in the pathogenesis of atherosclerosis, and the benefit of LDLc reduction in blood [1].

One central idea in ASCVD prevention is that the type and intensity of any preventive measure should be conditioned by the risk of developing ASCVD over time, especially in the short and medium term [2]. For LDLc reduction, the main international scientific societies recommend undertaking hygienic-dietary measures as the first step of lipid-lowering treatment in all patients, but also concomitantly initiating hypolipidemic treatment with potent statins in high-risk groups: subjects with very high concentrations of LDLc, subjects affected by severe genetic form of hypercholesterolemia, and patients who have already suffered an ASCVD event [3, 4]. In all of these cases, these guidelines recommend aiming to LDLc reduction > 50% with the use of high potency statins at high doses. The American College of Cardiology/American Heart Association guideline on the treatment of blood cholesterol to reduce ASCVD risk in adults, after analyzing the hypolipidemic efficacy of different statins in multiple clinical trials and performing head-to-head comparison among statins, classify them according to their hypolipidemic effect in statins of low, medium, and high potency. The latter group encompasses rosuvastatin at doses of 20 mg/day and 40 mg/day, and atorvastatin at doses of 40 mg/day and 80 mg/day. High potency statins allow LDLc reduction > 50% and for that intensity, a similar clinical benefit is assumed [3].

However, there are few observational reports and no randomized clinical trials in secondary prevention with recurrence of ASCVD as a major outcome with rosuvastatin, in contrast to atorvastatin [5–8]. So, the assumption of equivalent clinical benefit is based on their lipid-lowering capacity and the clinical benefit of rosuvastatin demonstrated in subjects in primary prevention. Given that subjects in secondary prevention have different clinical characteristics, such as the currently high prevalences of diabetes [9] and vascular revascularization [10] among them, and different concomitant medications, from subjects in primary prevention, it would be good to know whether the benefit of both statins is similar in secondary prevention in real life.

In order to analyze potential differences in the clinical response to atorvastatin and rosuvastatin in subjects in secondary ASCVD prevention, we have analyzed the clinical evolution of those subjects of the Dyslipemia Registry of the Spanish Society of Arteriosclerosis (SEA) who at the time of inclusion in the Registry had already suffered an ASCVD.

## Material and methods

This observational, retrospective, multicenter, national study in Spain was designed to determine potential differences between the use of atorvastatin and rosuvastatin in the ASCVD recurrence. The information was obtained from the Dyslipidemia Registry of the SEA [11]. This is an active online registry, where 50 certified lipid clinics distributed throughout all regions of Spain report cases of various types of primary hyperlipidemias. Anonymous clinical data collection in this registry was approved by a central ethical committee (Comité Ético de Investigación Clínica de Aragón, CEICA) and participants gave their written informed consent. Inclusion criteria were standardized in 5 training sessions before case recruitment. For patients in secondary prevention, the registry collects personal and family health history, anthropometry, physical examination, laboratory data, type of ASCVD, age at which the ASCVD event occurred, age at which statin treatment began, and history of lipid-lowering treatment [12]. Patients were eligible for inclusion in this study if they were 18 years of age or older with previous ASCVD at inclusion in the registry. ASCVD was defined as: coronary (myocardial infarction, coronary revascularization procedure, sudden death); cerebral (ischemic stroke with > 24-h neurological deficit without evidence of bleeding in brain imaging tests); peripheral vascular disease (PAD) (intermittent claudication with ankle arm index< 0.9, or arterial revascularization of lower limbs) or symptomatic or asymptomatic abdominal aortic aneurysm. Arterial hypertension was defined as systolic blood pressure ≥ 140 mmHg or diastolic blood pressure ≥ 90 mmHg or self-reported use of antihypertensive medication. Diabetes was defined as fasting plasma glucose ≥126 mg/dl, HbA1c ≥6.5%, or self-reported treatment with antidiabetic medications. Current smoking was defined as current smoking or having smoked in the last year. Former smoker was defined as a subject having smoked at least 50 cigarettes in his lifetime, but not having smoked in the last year.

### Follow up

The registry is designed so that at least once a year the data on the clinical evolution of the included patients are updated, with new anthropometric data, changes in risk factors or medication, and the appearance of new ASCVD events.

The main endpoint was defined as the occurrence of a new major ASCVD event composed of coronary heart disease (coronary death, acute coronary syndrome requiring hospitalization, or coronary revascularization due to angina), cerebrovascular (fatal and non-fatal stroke, or carotid revascularization), and peripheral arterial disease (arterial revascularization of the lower extremities).

Participants were divided according to the type of statins recorded at the time of inclusion in the registry. The statin documented in the registry represented the treatment for the follow-up years prior to the recurrence or censoring. Recurrent ASCVD event dates were collected and, in their absence, participants were censored at the date the follow-up data was obtained from the registry. Three different follow-up start-times were performed: starting from the time of inclusion in the registry (all participants had a previous event), starting from the time of first event if this occurred after 2005, and starting from the time of first event without date restriction.

### Statistical analysis

Data are expressed as mean and standard deviation for continuous variables with normal distribution and they were analyzed with the Student’s t test. Categorical variables are expressed as a percentage and analyzed by the × 2 test. The rates of adverse events up to the end of the follow-up were calculated by considering observed person-time and survival curves were created by Kaplan-Meier estimation, and the groups were compared by log rank test. The association between type of statin and ASCVD events was calculated using Poisson regression. Multivariable Poisson regression models were fitted including the covariates: age and sex (model 1), diabetes, hypertension, smoking status, body mass index (BMI), non-high-density lipoprotein (non-HDL) cholesterol, HDL cholesterol and ezetimibe use.

We conducted this study in accordance with the Declaration of Helsinki for the protection of the rights and welfare of people participating in biomedical research.

## Results

### Patient characteristics

**Fig. 1:**
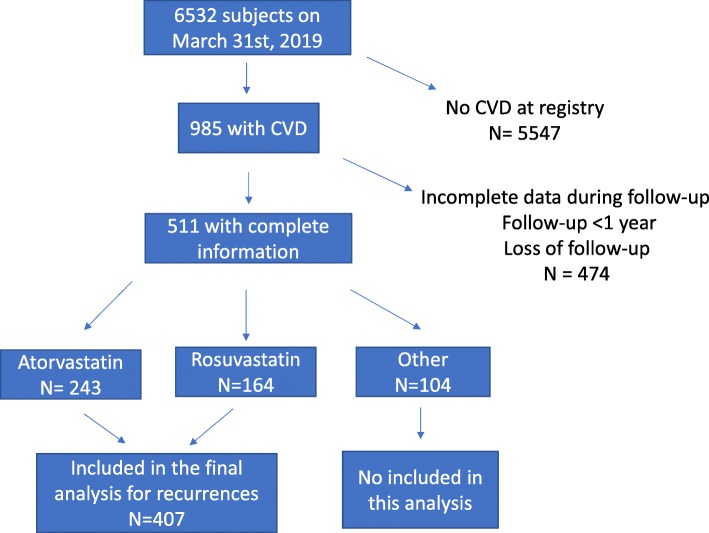
Recruitment process

In the registry, 985 subjects had had an ASCVD event at the time of their inclusion. On March 31st, 2019, follow-up data were evaluated and 475 subjects were excluded due to incomplete data, changes in the lipid-lowering drugs, follow-up less than 1 year, or loss to follow-up. There were no relevant clinical differences at registry between those included and excluded for the analysis (Additional file 3: Table S1). Only those subjects under continuous treatment with atorvastatin (*n* = 243) or rosuvastatin (*n* = 164) were included in this analysis (Fig. 1). Clinical characteristics at the moment of inclusion in the registry only differed in the gender proportion between both treatment groups (Table 1). In the atorvastatin group men were more frequent. At inclusion, the mean age in both groups was 61 years, there were no differences in body mass index, the prevalence of hypertension, diabetes, or smoking history between those patients on atorvastatin and rosuvastatin. The age of the first ASCVD and the type of ASCVD were also similar between the groups (Table 1).

**Table 1:** Clinical characteristic of subjects with CVD at inclusion in the Registry according to statin prescribed, and lipid values at diagnosis of dyslipidemia in the Lipid Clinic without lipid-lowering treatment and after lipid-lowering treatment recorded at inclusion in the Registry

Variables	Atorvastatin (*n* = 243)	Rosuvastatin (*n* = 164)	*P*
Gender (Male), % (n)	78.2 [190]	68.9 [113]	0.046
Age at inclusion, years	60.9 (11.1)	60.6 (9.9)	0.743
Body mass index, (Kg/m^2^)	28.9 (4.1)	28.6 (4.3)	0.595
ASCVD type (CHD/Stroke/PAD), %	77.3/14.5/7.0	79.3/12.2/5.5	0.460
Age first ASCVD event	51.7 (11.4)	51.1 (10.5)	0.561
Tobacco consumption, % (n)	18.9 [45]	15.5 [25]	0.461
Hypertension, % (n)	48.6 [118]	56.1 [92]	0.164
Diabetes, % (n)	30.9 [75]	31.7 [52]	0.943
Glucose, mg/dL	113.6 (33.5)	108.4 (32.4)	0.130
Age statin onset	48.4 (12.3)	49.4 (11.2)	0.477
Total cholesterol, mg/dl			
Pre-treatment	296.8 (102.6)	322.1 (111.1)	0.020
Post-treatment	172.9 (55.6)	182.6 (49.8)	0.065
HDL cholesterol, mg/dl			
Pre-treatment	45.2 (14.3)	46.6 (12.8)	0.285
Post-treatment	47.1 (13.8)	49.6 (11.6)	0.048
Non-HDL cholesterol, mg/dl			
Pre-treatment	251.6 (100.8)	275.5 (109.9)	0.027
Post-treatment	110.6 (39.9)	119.0 (49.5)	0.072
Triglycerides, mg/dl			
Pre-treatment	241.5 (291.3)	209.2 (229.4)	0.213
Post-treatment	161.5 (175.2)	160.2 (153.8)	0.938
Statin daily dose, mg/day	50.8 (24.7)	21.4 (9.6)	–
Ezetimibe use, % (n)	57.9 [140]	69.5 [114]	0.023

Values are percentage [count], mean (SD), as applicable. *ASCVD* Denotes arteriosclerotic cardiovascular disease, *CHD* Coronary heart disease, *PAD* Peripheral artery disease, *HDL* High-density lipoprotein

Total cholesterol and non-HDLc were higher before treatment in those subjects to whom rosuvastatin was prescribed. After treatment, HDLc has higher in the rosuvastatin group, and the differences in total cholesterol and non-HDLc were reduced to a level at which they did not reach statistical significance any more (Table 1). The mean dose of atorvastatin and rosuvastatin were 50.8 (24.7) mg/day and 21.4 (9.6) mg/day, respectively, corresponding to a medium dose of a high potency statin and they were equivalent with respect to their lipid-lowering efficacy. At the highest doses marketed in Spain (rosuvastatin 20 mg and atorvastatin 80 mg), there were no significant differences in the reduction of LDLc. The concomitant use of ezetimibe was very high among patients on atorvastatin, but higher in those patients on rosuvastatin, 57.9 and 69.5%, respectively (*p* = 0.023) (Table 1).

### Recurrences

**Fig. 2:**
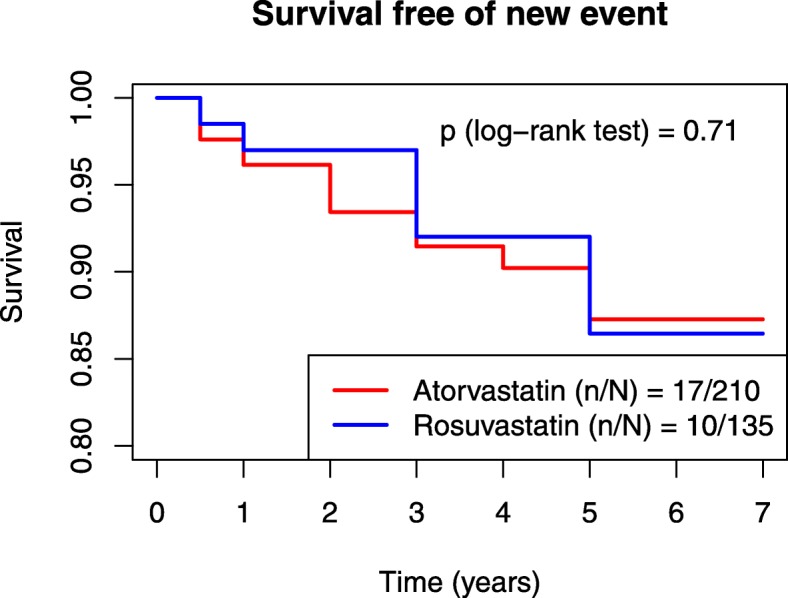
Cumulative incidence of the composite primary end point after inclusion in the registry

In the registry, among atorvastatin and rosuvastatin users, 89 recurrences of ASCVD after a first event were recorded (21.9%), of which 85.4% were coronary, 11.2% ischemic stroke, and 3.4% PAD; there were no hemorrhagic strokes or abdominal aortic aneurism surgery during evolution. At the inclusion of the subject in the registry, 345 participants had not suffered a recurrence yet. Thus 62 recurrences occurred before and 27 after inclusion on the registry. These 345 subjects accumulated 1050 person-years in a mean follow-up of 3 years. Event rates were 2.73 (95% CI: 1.63, 4.25) cases/100 person-years and 2.34 (95% CI: 1.17, 4.10) cases/100 person-years in the atorvastatin and rosuvastatin groups respectively (Fig. 2). There were no statistically significant differences between the two groups (crude, adjusted for age and sex, and for major cardiovascular risk factors Poisson models as described in methods). Subjects with recurrent ASCVD presented higher pre-treatment concentration of non-HDLc than those subjects without recurrences during the follow-up. All other clinical and biochemical variables did not differ between those who suffered recurrence and those who did not (Additional file 4: Table S2).

Among the patients in the registry with ASCVD (*n* = 407), 287 had their first episode in the last 15 years (year 2004 or later). Among them, 176 took atorvastatin at the time of inclusion in the registry and 111 subjects rosuvastatin. The Kaplan–Meier survival estimates for the end-point from the moment of the first event are shown in Additional file 1: Figure S1. Within an average follow-up of 7.5 years, 47 (16.4%) patients (28 in the atorvastatin group and 19 in the rosuvastatin group) suffered a second episode of ASCVD. Crude rates for this follow-up of 2154 person-years were 2.2 (95% CI 1.5, 3.1) and 2.2 (95% CI 1.3, 3.3) episodes per 100 person-years for the atorvastatin and rosuvastatin groups respectively, without finding statistically significant differences between the two groups (crude models, adjusted for age and sex, and for major cardiovascular risk factors as described in methods). Results did not differ when subjects with first events prior to 2004 (10.8 years mean follow-up) were included in the model (Additional file 2: Figure S2).

## Discussion

The present work shows that the recurrence of ASCVD events in the Registry of Dyslipemias of the SEA does not reveal relevant differences between those subjects in treatment with rosuvastatin or atorvastatin at the beginning of the follow-up. These results support the recommendation to use them as clinically equivalent in the secondary prevention of ASCVD when used at appropriate dose.

There are very limited studies that have analyzed the differences in clinical ASCVD events between statins. The Pravastatin or Atorvastatin Evaluation and Infection Trial (PROVE IT) analyzed the efficacy of atorvastatin 80 mg/day and pravastatin 40 mg/day in the prevention of cardiovascular recurrence after acute coronary syndrome. Atorvastatin provided greater protection against death or major cardiovascular events than pravastatin did. However, they used different doses, not equivalent with respect to their lipid-lowering potency, so their results support the use of powerful statins at high doses compared to statins of intermediate potency [12]. The Treating to New Targets (TNT) demonstrated that intensive lipid-lowering therapy with 80 mg of atorvastatin per day in patients with stable coronary disease provides benefit when compared with 10 mg of atorvastatin per day. Again, they used different doses, although with identical conclusions [5]. The IDEAL study, enrolled patients with a history of acute MI and were randomly assigned to receive a high dose of atorvastatin (80 mg/day) or simvastatin (20 mg/day). The intensive lowering of LDLc did not result in a significant reduction in the primary outcome of major coronary events, but did reduce the risk of other composite secondary end points and nonfatal acute MI [6]. Hence, there is high quality evidence that intensive lipid-lowering treatment with further reductions in LDLc produce further reductions in ASCVD [13], but there is no evidence of clinically meaningful differences between statins with the same lipid-lowering potency. In this study, we show that when using similarly powered statins in a high-risk population, rates of second events are similar, no matter the statin used.

An added value to our data is the high use of combined treatment in our registry. It must be kept in mind that these are specialized units and that many patients in the registry have severe primary dyslipidemias, many of them familial hypercholesterolemia. The fact that the results are similar in those subjects after adjusting for ezetimibe in the treatment gives more information about the clinical equivalence of both statins at equipotent doses.

Our study has several limitations. The main one is that it is an observational study and therefore subject to biases in the use of one or another statin. However, the data have been adjusted with the different potentially confounding variables without modifying the results. The follow-up of the subjects is also variable and it is not possible to analyze the therapeutic compliance during the follow-up. However, no differences in compliance between drugs have been described, so it does not seem to be a major problem. Finally, changes in treatment have not been covered during the period of follow-up previous to inclusion in the registry and some subjects have been able to change from atorvastatin to rosuvastatin and vice versa. This extreme is exceptional in the registry since the usual is the addition of ezetimibe in case of not achieving therapeutic goals, and the use of ezetimibe in both groups is well balanced [14].

## Conclusion

This observational and retrospective analysis of ASCVD recurrences does not find appreciable clinical differences between high doses of rosuvastatin and atorvastatin, and supports their use as clinically equivalent in secondary prevention of ASCVD.

## Supplementary information

**Additional file 1:** **Figure S1** Cumulative incidence of the composite primary end point in those 287 subjects with a first episode in the last 15 years (year 2004 or later).

**Additional file 2:** **Figure S2** Cumulative incidence of the composite primary end point in all subjects with a first ASCVD included in the Registry

**Additional file 3:** **Table S1** Clinical and biochemical differences at Registry between subjects with and without ASCVD recurrence after inclusion in the Registry

**Additional file 4:** **Table S2** Characteristics of patients with ASCVD at inclusion in the Registry according to inclusion or exclusion in the follow-up.

## Data Availability

The datasets used and/or analysed during the current study are available from the corresponding author on reasonable request.
