# Supplementary figures and images for: Comparative efficacy between atorvastatin and rosuvastatin in the prevention of cardiovascular disease recurrence
Source: Lipids Health Dis. 2019 Dec 11;18:216. doi: 10.1186/s12944-019-1153-x (PMC6905000; doi:10.1186/s12944-019-1153-x)

## Survival free of new event

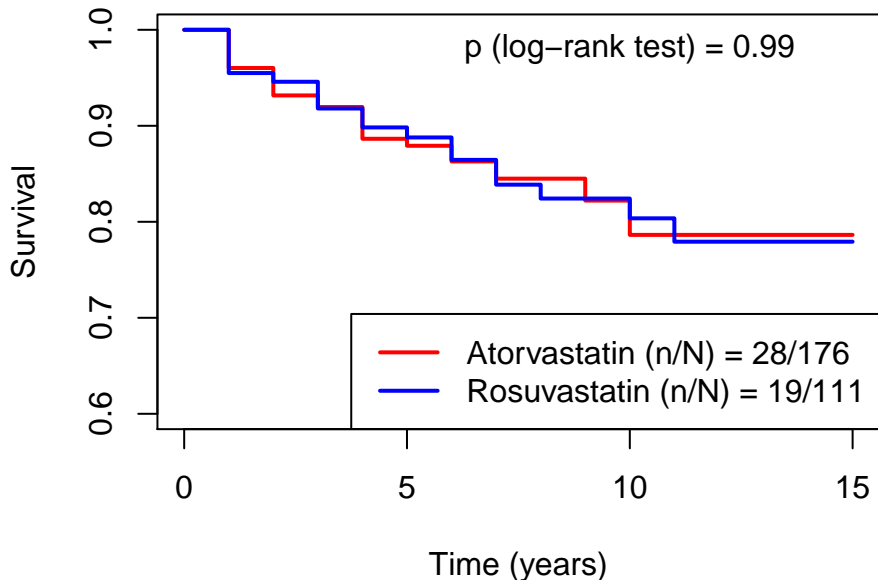

Supplement: Supplementary file 1 — Additional file 1: Figure S1 Cumulative incidence of the composite primary end point in those 287 subjects with a first episode in the last 15 years (year 2004 or later). [file 12944_2019_1153_MOESM1_ESM.pdf]

## Survival free of new event

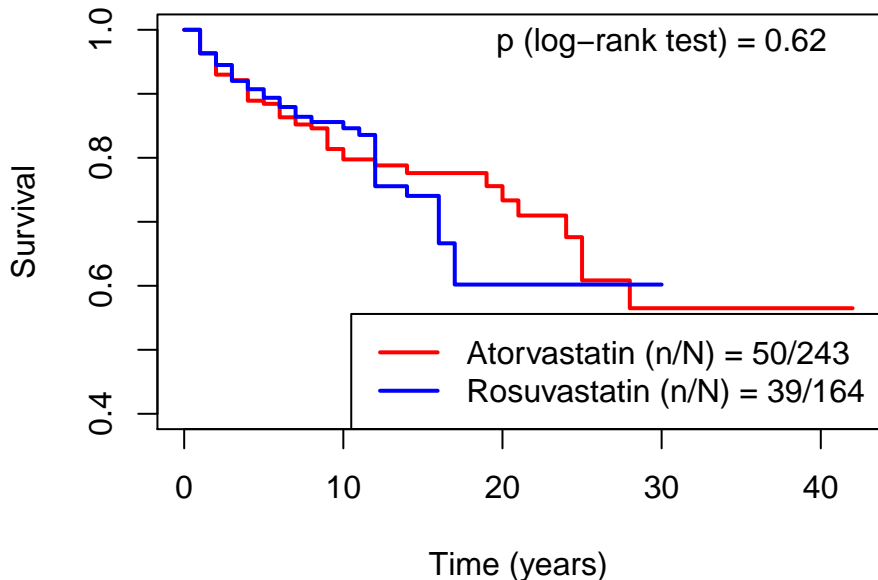

Supplement: Supplementary file 2 — Additional file 2: Figure S2 Cumulative incidence of the composite primary end point in all subjects with a first ASCVD included in the Registry [file 12944_2019_1153_MOESM2_ESM.pdf]
