# Supplementary material for: Comparative efficacy between atorvastatin and rosuvastatin in the prevention of cardiovascular disease recurrence
Source: Lipids Health Dis. 2019 Dec 11;18:216. doi: 10.1186/s12944-019-1153-x (PMC6905000; doi:10.1186/s12944-019-1153-x)
Supplement: Supplementary file 3 — Additional file 3: Table S1 Clinical and biochemical differences at Registry between subjects with and without ASCVD recurrence after inclusion in the Registry [file 12944_2019_1153_MOESM3_ESM.docx]

Supplemental Table 1. Clinical and biochemical differences at Registry between subjects with and without ASCVD recurrence after inclusion in the Registry

| **Variables** | With ASCVD recurrence (n=27) | Without ASCVD recurrence (n= 318) | *P* |
| --- | --- | --- | --- |
| Gender (Male) | 74.1 [20] | 73.9 [235] | 1.000 |
| Body mass index, (Kg/m^2^) | 28.4 (3.9) | 28.8 (4.1) | 0.684 |
| Age first ASCVD event, years | 52.2 (12.6) | 52.0 (10.6) | 0.941 |
| Age ASCVD recurrence, years | 62.7 (11.2) |  |  |
| Tobacco consumption, % (n) | 3.7 [1] | 17 [53] | 0.125 |
| Hypertension, % (n) | 55.6 [15] | 51.6 [164] | 0.844 |
| Diabetes, % (n) | 25.9 [7] | 32.4 [103] | 0.633 |
| Pre-treatment HDL cholesterol, mg/dl | 42.6 (10.9) | 46.3 (14.3) | 0.114 |
| Post-treatment HDL cholesterol, mg/dl | 50.1 (13.6) | 48.2 (13.4) | 0.506 |
| Pre-treatment non-HDL cholesterol, mg/dl | 314.1 (132.2) | 247.2 (94.4) | 0.016 |
| Post-treatment non-HDL cholesterol, mg/dl | 111.6 (38.7) | 114.2 (44) | 0.739 |
| Pre-treatment triglycerides, mg/dl | 234.9 (326.8) | 232.2 (280.2) | 0.967 |
| Post-treatment triglycerides, mg/dl | 202.3 (371.1) | 157.7 (146.7) | 0.541 |
| Age statin onset, years | 46.5 (14) | 49.1 (11.3) | 0.441 |
| Atorvastatin/Rosuvastatin at registry, % | 63.0/37.0 | 60.7/39.3 | 0.979 |
| Ezetimibe use at registry, % (n) | 66.7 [18] | 60.1 [191] | 0.639 |

Values are percentage [count], mean ± (SD), as applicable. ASCVD denotes arteriosclerotic cardiovascular disease; HDL, high-density lipoprotein.
