# Supplementary material for: Comparative efficacy between atorvastatin and rosuvastatin in the prevention of cardiovascular disease recurrence
Source: Lipids Health Dis. 2019 Dec 11;18:216. doi: 10.1186/s12944-019-1153-x (PMC6905000; doi:10.1186/s12944-019-1153-x)
Supplement: Supplementary file 4 — Additional file 4: Table S2 Characteristics of patients with ASCVD at inclusion in the Registry according to inclusion or exclusion in the follow-up. [file 12944_2019_1153_MOESM4_ESM.docx]

| **Variables** | Included (n= 511) | Excluded (n= 474) | *P* |
| --- | --- | --- | --- |
| Gender (Male), % (n) | 73.2 [374] | 71.5 [339] | 0.558 |
| Age at inclusion, years | 57.2 (11.2) | 58.0 (12.6) | 0.300 |
| Body mass index, (Kg/m^2^) | 28.7 (4.2) | 28.6 (4.5) | 0.595 |
| ASCVD type (CHD/Stroke/PAD), % | 76.9/13.8/1.0 | 76.5/14.5/1.3 | 0.445 |
| Age first ASCVD event | 51.7 (11.4) | 53.0 (12.3) | 0.081 |
| Tobacco consumption, % (n) | 17.8 [89] | 21.5 [96] | 0.114 |
| Total cholesterol, mg/dl | 297.8 (102.4) | 302.3 (117.5) | 0.522 |
| HDL cholesterol, mg/dl | 46.1 (14.1) | 45.6 (15.3) | 0.568 |
| Non-HDL cholesterol, mg/dl | 251.7 (100.5) | 256.8 (115-8) | 0.466 |
| Triglycerides, mg/dl | 237.0 (269.2) | 253.2 (436.4) | 0.481 |
| Age statin onset | 48.8 (12.3) | 49.1 (11.4) | 0.480 |
| Atorvastatin/Rosuvastatin % (n) / % (n) | 47.6 (243) / 32.1 (164) | 48.5 (230) / 30.1 (122) | 0.217 |

**Supplemental Table**. Characteristics of patients with ASCVD at inclusion in the Registry according to inclusion or exclusion in the follow-up.

ASCVD denotes atherosclerotic cardiovascular disease; CHD, coronary heart disease; PAD, peripheral artery disease; HDL, high-density lipoprotein
